# Cholera

**Published:** 1893-07

**Authors:** 


CHOLERA. ITS PROTEAN ASPECTS AND ITS MANAGEMENT. By Dr. G. Archie Stockwell, F. Z. S., Member New Sydenham Society, London. In two volumes of about 150 pages each. Price per volume, Paper, 25 cts.; cloth, 30 cts. Geo. S. Davis, Publisher, Detroit, Mich.

The work before us gives evidence of much care and study in its preparation. The author quotes from the leading writers of the day, and while he does not always agree with what they say, he gives some good reason for disagreeing. He treats the subject under the following heads: History, Epidemiology, Transmission dangers, Pathological discussion, Cholera characteristics, Reaction and Convalescence, Cholera Diarrhœa and Cholerine, Specific Pathology, Prophylaxis, past management of Cholera, general management during epidemics, management of pronounced Cholera, evidences of value of vagus treatment. Then follows the following appendices—"A" The history of European cholera and Asiatic cholera; "B" Asiatic cholera in Europe and America; report of State Board of Health of Tennessee; "C," The recent epidemic at Hamburg, by Prof. Max von Pettenkoffer. The author takes the ground that sanitation effects but little in the way of preventing the development and spread of cholera. He does not believe that the comma bacillus of Koch is the pathogenic agent of cholera but that it is a poison acting on the central nervous system and the influence thereby manifested upon and through the great sympathetic. He ridicules the old line of treatment, and proves by statistics that the treatment recommended by Alexander Harkin,—inhibition of the vagus, counter irritation, blisters, etc., behind the ears—is the only satisfactory one. The book is a very interesting one, written in a pleasing and pointed style, and in view of the threatened cholera invasion this year, will doubtless be much sought after and read. It is worthy of careful perusal. H.

